# Designing a Clean Label Fish Patty with Olive, Citric, Pomegranate, or Rosemary Extracts

**DOI:** 10.3390/plants9050659

**Published:** 2020-05-22

**Authors:** Lorena Martínez-Zamora, Gaspar Ros, Gema Nieto

**Affiliations:** Department of Food Technology, Nutrition and Food Science, Veterinary Faculty, University of Murcia, Regional Campus of International Excellence “Campus Mare Nostrum”. Campus de Espinardo, 30100 Espinardo, Murcia, Spain; lorena.martinez23@um.es (L.M.); gros@um.es (G.R.)

**Keywords:** antioxidant, citrus, fish patties, hydroxytyrosol, natural preservatives, *Punica granatum*, *Rosmarinus officinalis*

## Abstract

The natural functional ingredients derived from pomegranate (41.4% punicalagin), rosemary (5.8% carnosic acid and carnosol), hydroxytyrosol (7.3%), and citrus (55% hesperidin) fruits were combined separately with acerola (17% vitamin C) and essential oils rich in fatty acids (45% α-linolenic (ALA) and 40% docosahexaenoic (DHA)) provide a natural substitute of synthetic preservatives for fish patties, avoiding E-numbers on labels. Microbiological and physicochemical properties of the formulations were examined, sensory analysis was conducted, and changes in their shelf life due to storage for 14 days under chilled storage, adding these ingredients, were determined. The results obtained showed that the fish patties reported a high level of protein (14%), low fat (<2%), with a high contribution of phosphorus and selenium minerals, and higher levels of ALA up to 40% (in the case of rosemary extract (R)) and DHA by 30% (Ct), compared to the Control sample. The fish patties suffered microbiological, flavor, and odor spoilage and rapid lipid oxidation associated with rancidity. It can be said that the fish preparations have a duration of less than 7 days (between 4–6 days), except for the preparation with pomegranate extract (P) that has a longer life, from 7 to 11 days. Consequently, replacing synthetic additives by natural extracts offers a new clean label product with potential health benefits that resembles the commercial fish patties.

## 1. Introduction

Fish products are known to be highly nutritious and one of the fundamental supports of the Mediterranean diet. Fish generally has a low-calorie content and is an important source of proteins of high biological value (18–20%), vitamins, and minerals (Se, P, Fe, Mg, and K). Additionally, these kinds of products are rich in monounsaturated and polyunsaturated fatty acids (Ω3, 6, and 9), such as docosahexaenoic acid (DHA) and eicosapentaenoic acid (EPA), which make them play an important role in human nutrition [1]. Actually, its continued consumption not only contributes to normal heart function, but also maintains normal blood cholesterol levels, is essential for the development of the central nervous system during the first stages of life, and contributes to avoiding neurodegenerative chronic diseases [1].

According to FAO (Food and Agriculture Organization), global per capita fish consumption has increased from 9.9 kg in the 1960s to 20 kg in 2015. However, although worldwide fish consumption has increased in the last 50 years, in Spain it is quite the opposite. In Spain, the consumption of fish and fish products has decreased from 26.4 kg to 25.5 kg per person in the last 10 years. If these data are analyzed, it can be proved that consumption by people under 35 years of age is more reduced than the aforementioned reference, especially by children under 15 years old [2]. In addition, the Region of Murcia is the third Spanish autonomous community with the lowest fish consumption, preceded by the Canary Islands and the Balearic Islands. Therefore, new fish products are needed to encourage the consumption of this food group. In this sense, the development of healthy ready-to-eat products, such as fish burgers, based on our Mediterranean diet, is a good strategy to stimulate fish consumption, especially in young people [2]. However, these kinds of products are rich in synthetic additives to prolong their shelf-life, since fish muscle can be considered sterile when it is still alive, but its deterioration can occur rapidly after capture (enzymatic autolysis) and during subsequent stages of production, processing, and storage (lipid oxidation and bacterial growth) [3]. In parallel to the emerging market of clean label and functional animal origin products, it is interesting to study alternatives for the replacement of synthetic additives that have not already been studied [4,5,6,7,8].

The preservative effects of rosemary, hydroxytyrosol, or pomegranate extract (antioxidant and antimicrobial properties) are mainly due to the free radical scavenging ability and the inhibition of the activities of some enzymes [9,10]. However, few studies are available with the application of pomegranate, citric, and rosemary extracts for the extension of the shelf-life of fish.

In this field, Yerlikaya, Gokoglu, and Uran [11] studied the changes in the quality of anchovy burgers during refrigerated storage when these new formulas began to be found in supermarkets. Sehgal et al. [12] also studied the changes of microbiological growth and organoleptic quality of fish burgers prepared from carp, but they did not use natural antioxidants to preserve them. Salgado et al. [13] studied the use of films of sunflower protein enriched with essential oil of cloves and its application as preservatives in sardine burgers, but they did not apply these functional ingredients directly to the formula. López-Caballero et al. and Nowzari, Shábanpour, and Ojagh [14,15] studied the incorporation of a chitosan–gelatin blend as a coating for fish burgers and rainbow trout, respectively.

The main aim of this research was the development of fish patties exogenously enriched in Mediterranean antioxidant extracts obtained from rosemary, pomegranate, and olive tree, combined with essential fatty acids (α-linolenic (ALA) and docosahexaenoic (DHA) fatty acids) from linseeds and algae, respectively. For that, organoleptic, microbiological, oxidative, and autolytic changes of clean label fish patties for 14 days under refrigerated storage were extensively studied.

## 2. Results

### 2.1. Proximate Composition

The proximate composition of different samples of fish patties is shown in Table 1. As it can be observed, there were no significant differences among all the samples, which were made with frozen hake as the main ingredient (85%). Hence, samples presented 77.24–79.08% moisture, 2.72–3.29% ash, 14.84–15.99% protein, and 1.11–1.78% fat. Similar results were also obtained by Córser et al., Hleap et al., and Martí [16,17,18,19], who also used more than 80% fish for the elaboration of fish patties.

Significant differences were not found with regards to the results obtained from mineral analysis (Table 2) in the Control and reformulated fish patties. In a general view, Na was the most concentrated mineral, followed by, K, P, Mg, Fe, Zn, and Se. Additionally, the Control sample was the most abundant sample in K, Mg, and Zn, while the pomegranate (P) sample was the richest one in Fe and rosemary (R) in Na and P.

### 2.2. Shelf-Life Study

As it can be appreciated in Table 3, there were significant differences (*p* < 0.05) in pH analyses both on the day of analysis and between samples. Regarding the day of analysis, it is observed that all samples had a pH value of 6.8 at day 0 and a maximum peak was reached at day 7 of the study, decreasing again afterwards. In the case of the samples, it is observed that the P sample kept the pH levels constant on all the days of the study, while the rest of the samples increased the pH, even reaching pH 8 in the Control sample by the seventh day of the study.

**Figure 1:**
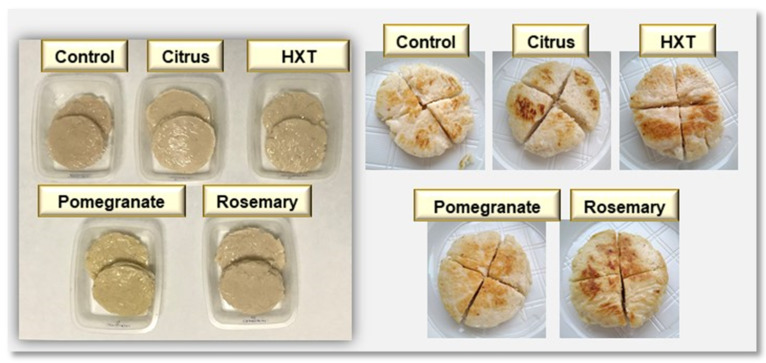
Photographs of raw or cooked fish patty samples.

Regarding color measurements, also presented in Table 3, there were significant differences (*p* < 0.05) between L*, a*, and b* coordinate results in all samples, indicating that the addition of extracts causes color modifications. The P sample was the one with the lowest values in the a* coordinate, followed by the hydroxytyrosol extract (HXT), citric extract (Ct), rosemary extract (R), and Control sample. However, the opposite occured at coordinate b*, where P had the highest content, followed by Ct, HXT, R, and Control. These differences can be observed visually, since the fish preparation with pomegranate extract presented a more intense orange-yellow coloration, which coincides with presented values of a* and b* (Figure 1).

Nevertheless, the results obtained from lipid and protein oxidation studies for 14 days are shown in Table 4, together with fish degradation results during the same period of time under chilled storage.

In the obtained thiobarbituric acid reactive substance (TBAR) values (lipid oxidation analysis) (Table 4), significant differences (*p <* 0.05) can be observed between the samples throughout the shelf-life study. Unless the values were slowly decreasing along the study, on day 14, the HXT, P, and R samples were less oxidized in comparison to Control and Ct, which reported the worst behavior.

With regards to protein oxidation, thiol groups analysis reported (Table 4) that the sample with the lowest protein oxidation throughout the shelf-life study was P, followed by HXT, Ct, R, and Control.

**Figure 2:**
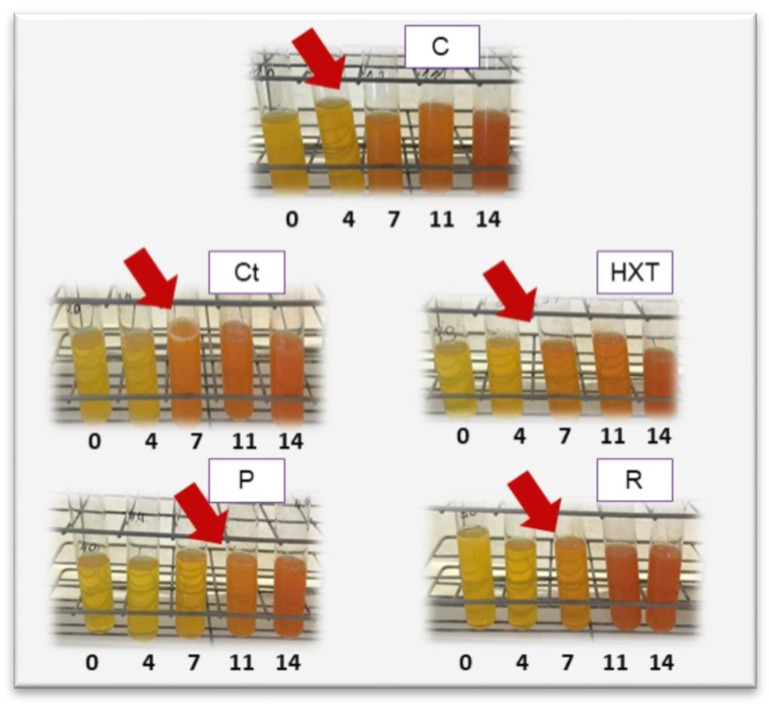
Ammonia determination with the Nessler colorimetric test in raw fish patty samples up to 14 days at 4 °C under simulated retail display conditions. Control sample (C); sample enriched in citrus (Ct); sample enriched in hydroxytyrosol (HXT); sample enriched in pomegranate (P); sample enriched in rosemary (R).

If we focus on the autolytic changes that happened in fish meat, as shown in Table 4, the results of trimethylamine (TMA) and total volatile basic nitrogen (TVB-N) can be obtained. Obtained results from the Nessler colorimetric test can be also visually appreciated in Figure 2. Regarding these data, there were no significant differences between the samples; there were only differences in the days of storage, increasing progressively to reach its maximum on day 14.

**Figure 3:**
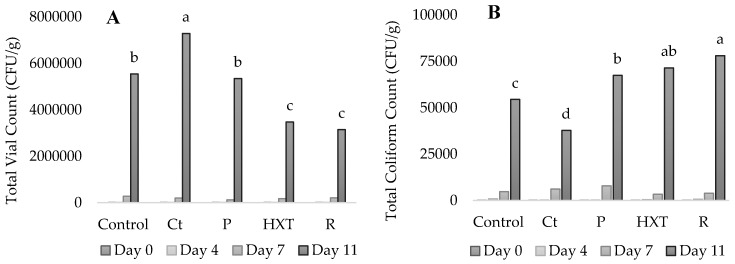
Evolution of microbiological growth during shelf-life study of fish patties. Total vial count (TVC; **A**). Total coliform count (TCC; **B**). Control (C); 200 ppm acerola + 200 ppm citric extract (Ct); 200 ppm acerola + 200 ppm hydroxytyrosol extract (HXT); 200 ppm acerola + 200 ppm pomegranate extract (P); 200 ppm acerola + 200 ppm rosemary extract (R). Different letters (a, b, c, d) in the same column indicate significant differences between samples (*p* < 0.05).

Figure 3 shows the antimicrobial capacity of the extract studied in fish patties at days 0, 4, 7, and 11 under refrigerated storage. As it can be appreciated in Figure 3A, total vial count (TVC) results in day 11 were lower in R samples, followed by HXT and P, while Ct reported significantly higher results (*p* < 0.05) than Control samples. Meanwhile, total coliform count (TCC) results in Figure 3B showed that both HXT and R extracts obtained the lowest results in comparison with the Control, P, or Ct samples on day 7 of storage.

Natural extract has been widely used as a natural antimicrobial compound, and, as it can be appreciated in Figure 3, in the present study, natural extracts acted as antimicrobial agents against TCC and TVC proliferation.

### 2.3. Volatile Compounds

**Figure 4:**
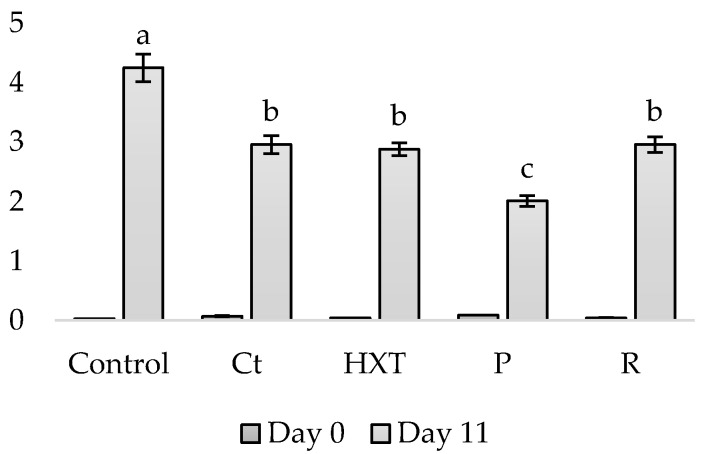
Evolution of hexanal (AU × 10^−6^ per g dry mater) (mean area ± standard deviation) of fish patties, elaborated with different natural extracts, after 11 days of at 4 °C under simulated retail display conditions. Control: 200 ppm acerola + 200 ppm citric extract (Ct); 200 ppm acerola + 200 ppm hydroxytyrosol extract (HXT); 200 ppm acerola + 200 ppm pomegranate extract (P); 200 ppm acerola + 200 ppm rosemary extract (R). Different letters (a, b, c) in the same column indicate significant differences between samples (*p* < 0.05).

The evolution of a volatile compound profile is shown in Table 5 and Figure 4. The presence of different kinds of volatile compounds and their changes during the shelf-life study can be due to several reasons, such as the incorporation of natural spices, lipid autooxidation, fish and microbiological degradation, or even the presence of pollution agents in the sea.

For instance, limonene and eucalyptol are volatile compounds related to the incorporation of acerola to all the samples (in case of limonene) and herbs, such as rosemary, from which comes eucalyptol. Hence, the presence of these compounds can be explained by the use of natural extracts as preservatives in fish patties.

### 2.4. Sensory Analysis

**Figure 5:**
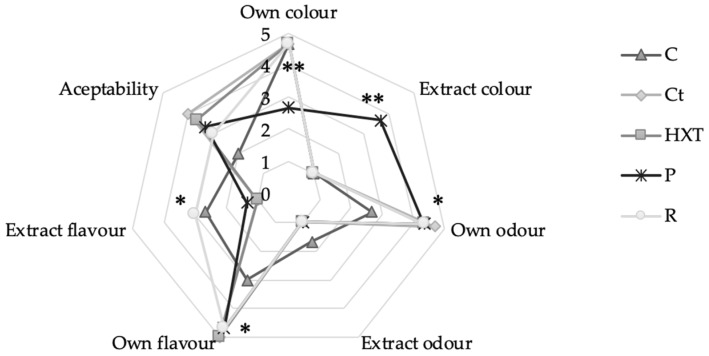
Sensory scores for fish patties stored in aerobic conditions for up to 14 days at 4 °C under simulated retail display conditions. Control sample (C); sample enriched in citrus (Ct); sample enriched in hydroxytyrosol (HXT); sample enriched in pomegranate (P); sample enriched in rosemary (R). * Significant differences *p* < 0.05. ** Significant differences *p* < 0.01.

Finally, the sensory analysis of fish patty samples was carried out at day 0 from elaboration in order to know the acceptability of these clean label fish products. The results obtained are shown in Figure 5.

Significant differences (*p* < 0.001) have been obtained in the score of the attribute of the proper color. In Control, Ct, HXT, and R, a hake color of its own was observed, while in P it was not. Similarly, “extract color” was appreciable only in the P sample (*p* < 0.001), being imperceptible in the Control, HXT, Ct, or R sample. This color change can also be appreciated in Figure 1, where the yellowness of the sample that incorporated the pomegranate extract (P) can be visually appreciated. In addition, in Table 3, a* values have been presented and show that P samples presented a yellowish tone different from the rest of the samples.

On the contrary, no significant differences have been observed between the different formulas in the parameters of proper odor and extract odor. Regarding flavor, differences have been observed in the taste of hake. Hence, the Control sample presented a spiced flavor (*p* < 0.05), while samples enriched in Ct, HXT, P, and R reported high scores of hake flavor (5, 5, 4.7, and 4.7, respectively). In this sense, a higher extract flavor score was obtained by R and Control (*p* < 0.05).

## 3. Discussion

Regarding proximate composition, the results obtained show that the incorporation of both phenolic-rich natural extracts and omega 3-rich essential oils did not affect the proximal content of fish patties. However, significant differences can be observed with regards to α-linolenic (ALA) and docosahexaenoic acid (DHA) content. For instance, although essential oils were added at the same percentage, samples that incorporated HXT or P into their formula presented 16–18% higher than samples elaborated with Ct or R extracts. The reason for this fact is that olive oil and pomegranate seeds are also natural sources of ALA [20,21] and these are the main ingredients for the elaboration of the cited extracts. On the contrary, the highest increase of DHA content was shown by Ct, followed by, R, HXT, and P. These variations between samples can be also due to lipid oxidation during the manufacturing of fish patties, which can degrade essential fatty acids to produce secondary products, such as malonaldehyde (MDA), which can be explained below by TBAR results. Additionally, the most important thing to note of this enrichment is that the fatty acid content of ALA and DHA was approximately improved with regards to the Control sample by 182 mg and 24.5 mg, respectively.

Regarding daily recommendations, the consumption of the studied manufactured fish products does not cover the necessary nutritional needs, but it can be said that their consumption provides 50% more minerals, as P or Mg, than the consumption of fresh hake, which has a concentration of 190 and 23 mg/100 g, respectively [22]. The inclusion of fish products such as this in a correctly balanced diet can be beneficial when helping to achieve the appropriate consumption requirements, especially for population groups with lower fish consumption, such as young people between 5 and 18 years of age [23].

Increases in pH in fish and fish products are an indicator of the accumulation of alkaline compounds, such as ammonia and TMA. This fact can be related to TMA, TVB-N, and qualitative ammonia results, which are shown below. In addition, autolytic changes and the release of these compounds can also derive from the action of microorganisms. However, the decrease of pH values from day 7 until 14 is an indicator of lactic acid bacteria growth. Once pH value decreases due to the bacteria growth, fish flavor turns to acid and sensory acceptance also decreases [24].

Parameters such as water retention capacity, collagen content, free water, and fat content affect L* coordinate, as does the addition of additives and other technological factors, such as cooking [25]. Therefore, obtaining differences in brightness (L*) between the preparations and control sample would be expected and made without phenolic rich extracts, because fish is a product that retains a large amount of water [25]. In addition, the red-green component is related to the presence of pigments, so its presence in foods, such as fish, in this case, will depend on the values of hemoglobin/myoglobin or punicalagin and carotenoids that may have been incorporated [26].

Lipid oxidation occurs after a chain reaction of free fatty acids, in which molecular oxygen participates and three phases can be distinguished: initiation (formation of the lipid radical), propagation (formation of the peroxyl radical), and termination (creation of oxidation secondary products responsible for the alterations associated with rancidity) [27]. Table 4 reported that P acts as the most antioxidant extract. This behavior, showed by the P sample, can be related with punicalagin and vitamin C content incorporated by pomegranate and acerola extracts. It has been demonstrated that phenolic compounds from pomegranate peels enhance its antioxidant activity [28]. Hence, this behavior can explain that 2000 ppm and 1000 ppm punicalagin-rich extracts (115.21 mg GAE/g of total phenolic content) have recently shown to reduce by 33% and 22% lipid oxidation, respectively, in sardine oil [29]. Similar results were also obtained by Ranucci et al. [30], who also described a reduction of lipid oxidation after the addition of pomegranate and citrus extracts (5 and 10‰) in vacuum-packaged sausages made from pork meat, emmer wheat, almond, and hazelnut.

Regarding antioxidant activity of HXT, in a previous study, Nieto et al. showed that TBAR values were 71% lower in chicken sausages containing HXT than in the Control samples. In addition, Kim et al. [31] showed that the combination of tomato powder and 1% olive oil resulted in the inhibition of lipid oxidation. The antioxidative activity of HXT extracts is mainly due to the radical scavenging activity and the metal ion chelation. Moreover, the external addition of rosemary extracts prevents lipid oxidation in red meat [32,33]. Rosemary extract contains bioactive compounds with antioxidant properties, such as isorosmanol, methylcarnosate, carnosic acid, rosmanol, carnosol, rosmarinic acid, and epirosmanol [34,35].

Bensid et al. and Özyurt et al. [36,37] reported that the limit of TBARs, beyond which fish samples have been considered unacceptable, is approximately 5 mg MDA/kg of fish flesh. This limit was not reached after 14 days of storage. It can be said that the hake used to elaborate fish patties contained low levels of fat (2% approximately), hence it could be expected that general samples did not present high values of lipid oxidation. Comparatively, lower TBAR values were obtained by Martí [16] in tuna and seaweed hamburgers, even though tuna is a fatter fish than hake, possibly because they were packaged in a modified atmosphere and vacuumed.

The fact that the Ct sample presents such lower values of lipid oxidation contrasts with higher values of protein oxidation reported. This may be due to the fact that the present phenolic in this extract (naringin, hesperidin) was powerful, acting on the agents responsible for this protein oxidation (transition metals, hydrogen peroxide). In addition, these results can be compared to previous exposed results by Martínez et al. [38], where the same extract, Ct, was tested in an oxidized pork meat model system and helped to control the protein oxidation in the presence of AAPH and AMVN agents.

In the same way, phenolics and potential antioxidant activity of the rest of the preservative compounds (HXT, P, and R) help to stabilize protein oxidation, reducing thiol loss from 9% (R) to 65% (P). It was then demonstrated that these extracts were more effective against protein oxidation than commercial preservatives (E-331, E-301, and E-262), which were added to the Control sample.

Furthermore, trimethylamine oxide (TMAO) is an osmoregulatory compound present in marine fish and its reduction is usually due to bacterial action; however, some fish species present in muscle tissue an enzyme (TMAO-ase) are able to break down TMAO into dimethylamine (DMA) and formaldehyde (FA). Visceral tissues have a high activity of the TMAO-ase enzyme; for this reason, it is really important to eviscerate and clean the fish before freezing. If this is not done, it has been demonstrated that the accumulation of FA produces hardening in hake muscle, which is also increased at high temperatures under frozen storage [39]. Unless autolytic changes precede the growth of microorganisms, this last part is the main cause of deterioration (25–30% of the origin of the loss of quality). This event is due the fact that fish have a high water content, free amino acids, and a high post-mortem pH level, and most marine species contain high levels of TMAO, which promotes bacterial growth (both Gram-positive and Gram-negative) [40]. When fish have been just captured, the muscle tissue is sterile. However, once post-mortem autolytic changes are carried out, skin and visceral bacteria start to grow and invade muscle tissue. In addition, fish deteriorates at different rates depending on storage conditions and the type of skin of the fish [40]. In the same way, this bacterial growth is responsible for the increase of volatile compounds, such as trimethylamine (TMA), volatile sulphurous compounds, aldehydes, ketones, and hypoxanthine, as well as basic volatile nitrogen compounds [41].

When TMA exceeds values of 15 mg TMA-N/100 g of fish while levels of TBV-N and NH_3_ are increasing, it is completely deteriorated, losing all its organoleptic quality, and rendering results unpalatable [42,43].

If we focus on autolytic changes that happen in fish meat, Table 4 provides the results of TMA and TVB-N. Regarding these results, there were no significant differences between the samples; there were only differences in the days of storage, increasing progressively to reach the maximum on day 14. However, if we carefully study the results, it can be observed how fish patties enriched with P extract showed better results than the R, HXT, Ct, and Control samples, in this order. Hence, the established quality limit of 1415 mg/100 g of fish is exceeded by the Ct and Control samples at day 11, while P and R samples still maintain TMA values under this level after 14 days. The P sample then acted as a better preservative agent in fish than HXT or R, with known antioxidative and protective capacities.

Similarly, the legal limits for TVB-N were established from 25 to 35 mg N/100 g. The only sample that reached day 11 without exceeding these values was the P sample. The increase of TVB-N and TMA was related to the microbiological activity, which coincides with the results obtained in a previous study [4], since on day 11 the microbial count increased considerably, being especially high after 14 days. In a comparative manner, in the study carried out by Martí [16], TVB-N and TMA did not exceed 20 mg TVB-N/100 g or 15 mg TMA/100 g due to the protective effect of packaging in a modified atmosphere. Nevertheless, in the present study, it was preferred to study the action of antioxidant agents in aerobic conditions. These data are also associated with ammonia content, the results of which have been shown in Figure 2. As appreciated, ammonia content is directly related to the obtained results of TMA and TVB-N, which justify the apparition of the orange color in the Control, Ct, and R samples. In this way, the P sample maintained the yellowness color after 11 days of refrigerated storage, which was 5 days more than the rest of the treatments.

The reduction of TMAO is also associated with the bacterial growth of *Photobacterium*, *Vibrio*, and *Shewanella putrefaciens*, but it is also carried out by Aeromonas and Enterobacteriaceae. During both anaerobic and aerobic growth, *S. putefraciens* uses the cycle of Krebs, where electrons are generated by a metabolic route (serine route) from carbon sources (acetate or succinate). From this route, other derivatives volatile compounds are produced, such as propanoic and malonic acid, from the glucose fermentation [44]. This behavior is also comparable to the release of toluene as a product of bacterial spoilage [45].

The production of TMA is carried out at the same time as the production of hypoxanthine, from autolysis of nucleotides. However, hypoxanthine can also be formed under the bacterial action of *Pseudomonas spp., S. putrefaciens*, and *P.phosphoreum* [46]. TMA conforms the major part of total basic volatile nitrogen (TBV-N). Hence, TMAO decreases in the fish, while TMA (MA and DMA) and TBV-N reaches the maximum level, due to the formation of ammonia (NH_3_) and other volatile amines. Once fatty acids and proteins are degraded, they are used as a substrate of anaerobic bacteria that produces high quantities of ammonia. Even after that, biogenic amines, such as histamine, putrescine, and/or cadaverine, are formed from decarboxylation of free amino acids as histidine, ornithine, and lysine, leading to a rotten smell [41]. This fact can also explain the apparent relation between the obtained results of TMA, through the shelf-life study (Table 4) and the methylamine levels (Table 5), as an indicator of the fish quality. The P sample was protected against fish degradation in 90% followed by R, HXT, Control, and finally, Ct.

The antimicrobial effectiveness of natural extract is due to the fact that they act as bacteriostatic compounds (inhibiting growth and extend shelf-life) instead of acting in terms of reducing initial microbial populations [47]. Shapira and Mimran [48] suggested that their antimicrobial effect might be due to the phenolic compounds. These compounds can disrupt the membrane of the cell affecting negatively to the functional properties of the cell [49]. Hence, it can be concluded that bioactive compounds (P, R, and HXT) act as antimicrobial agents, which is due to the phenolic compounds (hydroxytyrosol, punicalagin, carnosol, carnosic acid, rosmarinic acid) with known antimicrobial activity.

This antimicrobial activity was also shown in sausages made from pork meat, emmer wheat almond, and hazelnut after addition of pomegranate and citrus extracts (5 and 10‰) [30]. Furthermore, the antimicrobial effect of natural extracts has been previously observed by Del Nobile et al. [50], who reported how the inclusion of essential oils of grapefruit (100 ppm) and thymol (110 ppm) kept the microbial quality for 28 days of fish patties. Regarding studies reporting the bacteriostatic effect of medicinal plants and olive oil on sea bream, the study of Tassou et al. [51] reported a bacteriostatic effect on the flora autochthonous. In addition, Harpaz et al. [47] reported that the addition of oregano and thyme decreased the process of spoilage of Asian sea bream. On the same line, Mejlholm and Dalgaard [52] concluded that oregano oil (0.05% *v/w*) increased the shelf-life of cod fillets from 21 to 26 days at 2 °C. Moreover, the shelf-life of carp packaged for 12 days in air was extended with a dipping treatment of the fillets with thymol and carvacrol [53].

Another factor to take into account is the presence of arsenous acid as a contaminant, which other authors have already found in fish [54,55,56]. This is normal for marine fish and for the presence of arsenic in our oceans. However, marine arsenic represents a low risk to humans from fishery products, since it is not converted to toxic inorganic arsenite [55]. In this way, fish is also rich in other contaminants, such as furans, benzenes, and their derivatives [57,58], whose obtained results in the present study did not present significant differences.

Apart from that, other volatile compounds related to lipid autooxidation are also shown in Table 5 and Figure 4. These obtained values of hexanal, 1-butanol, and 2-nonanone can be associated with TBAR values.

Figure 4 shows evolution of hexanal. Hexanal values ranged from 0.1 mg/kg, on day 0, to 4.25 AU × 10^−6^ per g dry mater on day 11 of storage in control samples (compared to 2.96, 2.94, 2.88, and 2.01 AU × 10^−6^ per g dry mater in Ct, R, HXT, and P, respectively). These values are similar to those reported by Ahn et al. [59], in cooked ground beef with a concentration of 2.22 after 3 days storage in air, and the results reported by Brunton et al. [60], with hexanal values in cooked turkey of 4.01 l/g at day 6. Differences in the mean hexanal levels between C and fish patties with natural extracts were significant lower (*p* < 0.05) on day 11. On day 11, R showed 32% lower hexanal values than the Control samples, meaning that the Rosemary extracts were the best extracts that improved lipid stability of the fish patties. Similar to our results, Mi et al. [61] reported lower hexanal content in in red drum fillets, added with 6-gingerol at day 15 of storage, compared with the Control group.

The changes in the hexanal content over 11 days of storage were similar to those observed for the TBAR values, because hexanal is a good indicator of lipid oxidation and has been used as an indicator of flavor acceptability and oxidative stability [62].

The reason for the hexanal predominance over the other volatile compounds is attributed to the variety of its synthesis pathways [63]. In this sense, Kerler and Grosch [64] concluded that hexanal can be generated through the degradation of 2,4-decadienal and from degradation of arachidonic, linoleic, and oleic acid acids. In addition, these authors reported that hexanal showed a low-flavor threshold and a high rate of formation, resulting in unpleasant notes of flavor [65]. Moreover, previous studies have reported the relationship between off-flavor scores and hexanal [62,63].

A sensory analysis of reformulated fish patties gives a better definition of the appeal of the new product on the population and their intention to purchase, which greatly influences market trends for this type of product. In this case, obtained results from sensory analysis are due to the presence of phenolic and volatile compounds with intense flavor, such as rosmarinic acid, carnosic, and carnosol. Additionally, the C sample showed a notorious extract flavor produced by the commercial mix used to elaborate this batch.

It can be observed that the most accepted hake preparations were those of the Ct and HXT samples, followed by P, R, and C, which were the worst scored, due to the presence of a lot of synthetic flavorings in the commercial mix that gave a flavor completely different from fish. Comparable results were shown by Hleap Borrero et al. [17] on surimi preparations. Moreover, Ranucci et al. [30] also showed an improvement of sensory perception after the addition of 5% and 10‰ pomegranate and citrus extracts in a vacuum-sealed, post-packaged, pasteurized cooked sausage made with meat and a high proportion of non-meat ingredients (emmer wheat, almond, and hazelnut). Hence, it can be concluded that citrus (Ct) extract, incorporated as preservatives to fish patties, can be highly accepted; however, in this case, pomegranate (P) was reported to be a valuable strategy to enhance the shelf-life by controlling the microbial and oxidation spoilage during refrigerated storage while maintaining the sensory perception, as well as widely used commercial mixes (C). Additionally, several studies have reported the preservative effect of natural extract in foods [66,67,68]. After the shelf-life study, it can be said that all samples had a maximum consumption date of 7 days, except the P sample, which reached 14 days under refrigerated storage. In addition, these samples were better sensory evaluated than the Control sample. Hence, the P sample was the best qualified in all the analyses carried out.

## 4. Materials and Methods

### 4.1. Natural Oils and Extracts

The plant extracts (powder) used were Citrus extract (Ct), obtained from *Citrus sinensis,* with 55% of hesperidin; hydroxytyrosol (HXT), obtained from vegetation waters of olive (*Olea europaea*) tree with 7.26% pure bioactive compound, pomegranate (P) extract, obtained from peels of *Punica granatum* with main bioactive compounds (41.38% of total punicalagins)*,* and rosemary extract (R: *Rosmarinus officinalis* L. Leaves), which contained 5.76% of diterpenes (carnosic acid and carnosol). These extracts were supplied by Nutrafur-Frutarom S.A. (Alcantarilla, Murcia, Spain). Acerola (*Malpiguia emarginata*) extract, which contained 17% vitamin C, was used in all the samples and was supplied by Ferrer Alimentación S.A. (Barcelona, Spain). All extracts were diluted in water.

Essential oils ECOFLAX ^®^ and BIOMEGA TECH ALGAE ^®^ were supplied by BTSA (Alcalá de Henares, Madrid, Spain). ECOFLAX ^®^ was obtained from linseeds with 45% α-linolenic fatty acid (ALA), while BIOMEGA TECH ALGAE ^®^ was elaborated from algae with 40% docosahexaenoic (DHA) fatty acid.

### 4.2. Fish Patty Manufacture and Sampling

Hake fillets (*Merluccius capensis, Merluccius paradoxus*) (Pescanova España S.L.U.) were bought in a local supermarket and thawed in refrigeration for 24 h before elaborating the fish burgers. Fish was minced for 2 min, mixing all the ingredients of each one of five batches (Table 6). The Control sample was elaborated using a commercial mix composed by vegetables fibers, salt, potato starch, stabilizer (processed Eucheuma seaweed (PES) E-407a), sodium acetate E-262), acidity correctors (sodium citrate E-331), antioxidant (sodium ascorbate E-301), spice extracts, and spices. Fish patties were formed (50 g) and packed in aerobic conditions. Samples were stored at 4 °C until analysis at days 0, 4, 7, 11, and 14. A total of 50 fish patties were formed for each batch (10 fish patties per day of analysis), and analyses were carried out in triplicate.

### 4.3. Proximate Composition

Fish patty samples were analyzed for their moisture, ash, lipid, and total protein contents, according to AOAC methods at day 0 after elaboration. The mineral content of each sample was measured by plasma spectroscopy (ICP-OES) using an ICAP THERMO DUO 6500 computer, according to AOAC methods [69]. Analyses were carried out in triplicate.

#### Fatty Acids Profile

For the analysis of the fatty acids profile of each sample, firstly, an extraction of the fat following the method described by Folch et al. was carried our [70]. For that, total lipids were extracted from fish patty samples mixed with chloroform/methanol (2:1, *v/v*) and using a rotary evaporator (Laborota 4002: Control, Heidolph). After methylation with 2 N methanolic potassium, samples were injected in the Agilent gas chromatograph, model 7890 B. The column was the HP-5, 30 m, 0.320 mm., 0.25 µm, with a column flow of 1.4 ml/min; split 1:1 with injector at 260 °C; oven at 125 °C up to 145 °C at a rate of 8 °C/min. It remained at 145 °C for 26 min, rising to 220 °C at 2 °C/min and remained at 220 °C for 15 min. The FID detector was at 260 °C.

The identification of fatty acid methyl ester was performed by the comparison of retention times of fatty acid patterns of reference methyl esters, ALA (C18:3n3) (CAS: 463-40-1) and DHA (C22:6n3) (CAS: 2566-90-7) (Sigma-Aldrich, S.A. Madrid, Spain). The methyl esters of fatty acids were quantified using an internal standard (undecanoic acid methyl ester). The results were expressed as the percentage of methyl esters of each fatty acid. Analyses were carried out in triplicate.

### 4.4. Shelf-Life Study

pH was measured using Crison GLP21 equipment (Crison Instruments S.A., Barcelona, Spain) at 0, 4, 7, 11, and 14 days in triplicate (ISO 2917:1999).

Color was measured using a Konika Minolta CR 400 chromameter, standardized using a calibration plate (Minolta Camera Co., Osaka, Japan) in sextuplicate. Lightness (L*), green-red chromaticity (a*), and blue-yellow chromaticity (b*) were measured according to the CIELab system. The color coordinates were analyzed at 0, 4, 7, 11, and 14 days.

Thiobarbituric acid reactive substance (TBAR) content, directly related to lipid oxidation, was measured following the method described by Wang and Xiong [71] with some modifications. For that, 2.0 ± 0.1 g of sample were weighed and mixed with 1.5 ml of 1% thiobarbituric acid (TBA) solution and 8.5 ml 2.5% trichloroacetic acid-hydrochloric acid (TCA-HCl). This mix was then placed in a water bath at 100 °C for 30 min. After cooling to room temperature, 5 ml of mixtures (with no fish) were mixed with 5 ml of chloroform and centrifuged at 3500 rpm for 10 min. After that, the absorbance of the supernatant (colored) phase was read at 532 nm using a UV2 spectrophotometer (Pye Unicam Ltd., Cambridge, United Kingdom). The TBAR value, expressed as mg of malonaldehyde (MDA) per kg of fish patty sample, was calculated using the next formula.

TBAR (mg MDA/kg) = (Abs532/W) × 9.48(1)

Where Abs532 was the absorbance at 532 nm and W was the weight of the sample (g). The analyses were repeated in triplicate at 0, 4, 7, 11, and 14 days of chilled storage.

Microbiological growth of total vial count (TVC) and total coliform count (TCC) were determined. The analyses were repeated in triplicate at 0, 4, 7, and 11 days of chilled storage and expressed in cfu/g. Rapid *E. Coli* to determine TCC and PCA to determine TVC were used. Peptone water (OXOID, Ltd. CM0087 Basingstoke, Hampshire, United Kingdom) was used to make the dilutions. Plates were incubated for 24 h at 37 °C for TCC and 48 h at 37 °C for TVC.

Protein oxidation was related to the thiol loss. The concentration of the thiol groups was determined spectrophotometrically after derivatization by Ellman’s reagent, 5, 5-Dithiobis (2-nitrobenzoic acid) (DTNB) [72], following the method described by Nieto, Martínez, and Ros [4]. For that, 2.0 ± 0.1 g of each sample was mixed with 50 ml 5% sodium dodecyl sulphate (SDS) (pH = 8.0), dissolved in 0.10 M Tris(hydroxymethyl)aminomethane (TRIS) buffer (pH = 8.0), which was placed in a water bath at 80 °C for 30 min, and then centrifuged at 3000 rpm for 20 min. After that, the supernatants were filtered, and the protein concentration was determined spectrophotometrically at 280 nm. The absorbance corresponding to the thiol concentration in the samples was correlated with Abs_412-after DTNB_ – Abs_412-before DTNB_ – Abs_412-blank_. The analyses were carried out in triplicate at 0, 4, 7, 11, and 14 days after elaboration.

The degree of spoilage in fish was characterized by the production of TMA or other volatile bases. For the measurement of trimethylamine (TMA) content, it was extracted, according to the official method of AOAC 971.14 [66], adding 50 ml of 7.5% TCA (trichloroacetic acid) to the sample (25.0 ± 0.1 g), crushing, and obtaining a filtrate (4 ml). Subsequently, the reaction with 5 ml of 0.02% picric acid resulted in a colored complex, which was proportional to the TMA content of the sample, which was measured by its absorbance at 410 nm. The results were calculated using a TMA calibration curve and expressed as mg TMA/100 g. For that, the next formula was used.

mg TMA/100 g = (mg(N-TMA) × 50 × 100)/4 × W(2)

Where mg(N-TMA) was calculated using the calibration curve and W was the weight (g) of the sample. The analyses were repeated in triplicate at 0, 4, 7, 11, and 14 days of chilled storage.

The analytical procedure for the determination of total basic volatile nitrogen (TVB-N) was based on the reference method described in Regulation (EC) No 2074/2005. To extract the volatile nitrogenous bases, 10.0 ± 0.1 g of the sample was mixed with 90 ml of 6% perchloric acid and filtered. Then, 50 mL of filtrate was distilled by a previous addition of 10 ml of 20% NaOH. Finally, the nitrogenous bases were determined by titration with 0.01 N HCl until pH = 5.0. The results were calculated using the next formula

mg TVB-N/100 g = ((V_1_ − V_0_) × 0.14 × 2 × 100)/W (3)

Where V_1_ was the volume (ml) of 0.01 N HCl used per sample, V_0_ was the volume (ml) of 0.01 N HCl used per blank (using 50 ml of 6% perchloric acid with no sample), and W was the weight (g) of the sample. The analyses were repeated in triplicate at 0, 4, 7, 11, and 14 days of chilled storage.

A qualitative analysis, the Nessler color test, was also performed to determine the ammonia (NH_3_) content of the samples, which increased as the quality of the fish decreased (AOAC 973.49) [66]. For that, 2.0 ± 0.1 g of the sample was mixed with 5 ml of 5% TCA (trichloroacetic acid), left for 15 min, and centrifuged at 4000 rpm for 10 min. Then, 10 ml of filtered extract were mixed with 5 ml of Nessler reagent (potassium iodomercurate). After the reaction, the reddish colored complex of iodamide showed the quality of the samples. The analyses were repeated in triplicate at 0, 4, 7, 11, and 14 days of chilled storage.

For the measurement of the profile of volatile compounds, 5 g of fish patty samples were placed in glass vials, and these compounds were extracted using solid-phase microextraction (SPME: Supelco, Bellefonte, PA, USA, containing a fused-silica fiber). Volatile compounds were analyzed in triplicate in all the fish burger samples at 0 and 11 days. Compounds were identified by comparing their mass spectra with those contained in the NIST05 (National Institute of Standards and Technology, Gaithersburg) library. Results were expressed as a volatile compound profile (M ± s.e.).

### 4.5. Sensory Analysis

The tasting room for sensory evaluation was air-conditioned and free of disturbing factors. The fish patty samples were cooked and cut in four pieces, as presented in Figure 1. Sensory analysis was carried out at the same day of elaboration.

A total of 10 panelists were trained, following the ISO guide (2012). Samples were coded with three random digits and were presented individually to the panelists. Mineral water and stick breads were provided for mouth rinsing between samples. The attributes measured for the color, odor, and taste characteristics were: Own Color, Extract Color, Brightness, Homogeneity, Own Odor, Rancid Odor, Extract Odor, Own Flavor, Salty Flavor, Rancid Flavor, Extract Flavor, Hardness, Chewability, Juiciness, Granularity, and Acceptance.

### 4.6. Statistical Analysis

Data were analyzed with the statistical package SPSS 15.0 (Statistical Package for the Social Science for Windows (IBM, Armonk, New York, USA)), using a two-way ANOVA, taking into account variables of time and treatment. A value of *p* < 0.05 was considered statistically significant, using the Scheefe post-hoc test. Pearson’s correlation was applied to test correlations between groups.

## 5. Conclusions

Pomegranate and Rosemary extracts can be used to replace synthetic additives in fish patties when formulating clean label products; as a natural product, it allows clean labelling, avoiding E-numbers. When preparing fish patties using these ingredients, it is found that shelf-life of fish patties was extended up to 11 days, with mechanisms that slow down microbiological spoilage and autolytic changes (degradation of non-protein nitrogen (NPN) components), as well as lipid or protein oxidation, keeping the same sensorial high acceptability for panelists with no detection of abnormal flavors (smell or taste). Therefore, fish patties with functional ingredients, such as pomegranate, could be an interesting strategy by the food industry in order to reduce synthetic additives.

## Figures and Tables

**Table 1:** The proximate composition (g/100 g) of fish patty samples enriched in phenolic compounds and α-linolenic (ALA; C18:3n3) and docosahexaenoic acid (DHA; C22:6n3) fatty acids.

Samples	Moisture (%)	Ash (%)	Protein (%)	Fat (%)	ALA (%)	DHA (%)
Control	78.99 ± 1.02	2.87 ± 0.04	14.84 ± 0.63	1.11 ± 0.06 ^c^	0.05 ± 0.00 ^c^	0.09 ± 0.00 ^d^
Ct	79.08 ± 1.03	2.72 ± 0.12	15.24 ± 0.42	1.46 ± 0.05 ^b^	11.20 ± 1.06 ^a^	1.93 ± 0.09 ^a^
HXT	77.24 ± 0.83	2.98 ± 0.16	15.99 ± 0.41	1.62 ± 0.04 ^a^	12.50 ± 0.79 ^a^	1.34 ± 0.04 ^c^
P	77.52 ± 1.25	3.29 ± 0.05	15.39 ± 0.54	1.67 ± 0.09 ^a^	11.80 ± 0.95 ^a^	1.22 ± 0.08 ^c^
R	77.63 ± 0.92	3.14 ± 0.04	15.87 ± 0.34	1.78 ± 0.10 ^a^	9.48 ± 0.52 ^b^	1.51 ± 0.05 ^b^

Control: 200 ppm acerola + 200 ppm citric extract (Ct); 200 ppm acerola + 200 ppm hydroxytyrosol extract (HXT); 200 ppm acerola + 200 ppm pomegranate extract (P); 200 ppm acerola + 200 ppm rosemary extract (R). α-linolenic acid (ALA); docosahexaenoic acid (DHA). The results of ALA and DHA content were expressed as percentage of methyl esters of each fatty acid of the total fat percent. Mean ± standard error (M ± s.e.). Different letters in the same column (a, b, c, d) indicate significant differences between samples (*p* < 0.05).

**Table 2:** Mineral content (m ± s.e.) (mg/100 g) of fish patties and RDA percentage that supposes consumption of 100 g per day.

Samples	Fe	K	Mg	Na	Se	Zn	P
Control	0.7 ^a^ 5%	534 ^a^ 15.3%	68 ^a^ 20%	409 ^e^ 20.5%	0.03 45%	0.4 ^a^ 2.6%	214 ^d^ 30.6%
Ct	0.5 ^ab^ 3.9%	288 ^e^ 8.2%	39 ^d^ 11.5%	651 ^d^ 32.5%	0.02 28.8%	0.2 ^c^ 1.6%	209 ^e^ 29.9%
HXT	0.3 ^b^ 1.8%	349 ^b^ 10%	49 ^b^ 14.4%	751 ^c^ 37.6%	0.022 35.8%	0.3 ^b^ 1.7%	281 ^b^ 40.1%
P	0.7 ^a^ 5.1%	305 ^c^ 8.7%	42 ^c^ 12.4%	818 ^b^ 41%	0.02 25.6%	0.2 ^e^ 1.4%	272 ^c^ 38.9%
R	0.4 ^b^ 3.5%	300 ^d^ 8.5%	40 ^cd^ 11.8%	846 ^a^ 42%	0.02 30.2%	0.2 ^d^ 1.5%	292 ^a^ 41.8%
RDA (mg/day)	10–18	3500	330–350	<2000	0.055–0.070	15	700

Control: 200 ppm acerola + 200 ppm citric extract (Ct); 200 ppm acerola + 200 ppm hydroxytyrosol extract (HXT); 200 ppm acerola + 200 ppm pomegranate extract (P); 200 ppm acerola + 200 ppm rosemary extract (R). Recommended dietary allowance (RDA); Mean ± standard deviation (M ± s.d.). Different letters in the same column indicate significant differences between samples (*p* < 0.05).

**Table 3:** pH and color (CIELab) (m ± s.e.) evolution of fish patty for 14 days under refrigerated storage.

Sample	Days *Refrigerated Storage* at 4 °C				
	0	4	7	11	14
**pH**					
Control Ct HXT P R	6.8 ± 0.0 ^az^ 6.8 ± 0.0 ^az^ 6.8 ± 0.0 ^az^ 6.8 ± 0.0 ^bz^ 6.8 ± 0.1 ^abz^	7.0 ± 0.0 ^ayz^ 7.1 ± 0.0 ^ayz^ 7.0 ± 0.1 ^ayz^ 6.8 ± 0.1 ^byz^ 6.8 ± 0.0 ^abyz^	8.0 ± 0.1 ^ax^ 7.6 ± 0.0 ^ax^ 7.4 ± 0.0 ^ax^ 6.8 ± 0.0 ^bx^ 7.0 ± 0.1 ^abx^	7.3 ± 0.0 ^axy^ 7.2 ± 0.0 ^axy^ 7.3 ± 0.0 ^axy^ 6.7 ± 0.0 ^bxy^ 7.3 ± 0.0 ^abxy^	7.1 ± 0.0 ^axyz^ 7.2 ± 0.0 ^axyz^ 7.2 ± 0.1 ^axyz^ 6.6 ± 0.1 ^bxyz^ 7.1 ± 0.0 ^abxyz^
Colour *Parameters*: L*					
Control Ct HXT P R	72.3 ± 0.3 ^by^ 80.0 ± 1.2 ^ay^ 87.8 ± 2.3 ^ay^ 82.3 ± 0.9 ^ay^ 81.9 ± 1.1 ^ay^	65.0 ± 0.8 ^bz^ 73.9 ± 1.3 ^az^ 73.6 ± 0.8 ^az^ 70.9 ± 0.5 ^abz^ 71.7 ± 0.4 ^az^	62.8 ± 0.6 ^bz^ 72.2 ± 0.4 ^az^ 71.8 ± 0.3 ^az^ 70.4 ± 0.5 ^az^ 70.2 ± 0.4 ^az^	64.7 ± 0.5 ^bz^ 72.3 ± 0.5 ^az^ 72.8 ± 0.7 ^az^ 69.5 ± 0.8 ^az^ 70.3 ± 0.6 ^az^	64.5 ± 0.2 ^bz^ 73.0 ± 0.6 ^az^ 72.5 ± 0.3 ^az^ 69.2 ± 0.4 ^az^ 69.8 ± 0.3 ^az^
a*					
Control Ct HXT P R	3.5 ± 0.0 ^az^ 2.4 ± 0.1 ^bz^ 1.9 ± 0.1 ^bz^ 0.7 ± 0.1 ^cz^ 2.6 ± 0.0 ^bz^	3.7 ± 0.1 ^ayz^ 2.4 ± 0.1 ^byz^ 2.1 ± 0.0 ^byz^ 1.4 ± 0.1 ^cyz^ 2.7 ± 0.0 ^byz^	4.7 ± 0.1 ^ay^ 3.4 ± 0.1 ^by^ 3.0 ± 0.1 ^by^ 1.9 ± 0.1 ^cy^ 3.3 ± 0.0 ^by^	4.2 ± 0.1 ^ayz^ 3.4 ± 0.8 ^byz^ 2.9 ± 0.0 ^byz^ 2.2 ± 0.2 ^cyz^ 3.3 ± 0.1 ^byz^	3.7 ± 0.1 ^ayz^ 2.4 ± 0.1 ^byz^ 2.4 ± 0.1 ^byz^ 1.5 ± 0.0 ^cyz^ 2.8 ± 0.0 ^byz^
b*					
Control Ct HXT P R	11.4 ± 0.1 ^c^ 11.9 ± 0.4 ^b^ 11.9 ± 0.4 ^c^ 18.7 ± 0.4 ^a^ 12.1 ± 0.4 ^c^	11.3 ± 0.2 ^c^ 13.7 ± 0.1 ^b^ 11.1 ± 0.2 ^c^ 17.0 ± 0.3 ^a^ 11.1 ± 0.7 ^c^	12.2 ± 0.3 ^c^ 14.8 ± 0.1 ^b^ 13.0 ± 0.3 ^c^ 16.2 ± 0.2 ^a^ 11.5 ± 0.1 ^c^	12.1 ± 0.2 ^c^ 14.6 ± 0.4 ^b^ 12.4 ± 0.3 ^c^ 16.5 ± 0.3 ^a^ 10.6 ± 0.5 ^c^	11.0 ± 0.1 ^c^ 13.8 ± 0.3 ^b^ 12.4 ± 0.1 ^c^ 15.7 ± 0.2 ^a^ 11.2 ± 0.2 ^c^

Control: 200 ppm acerola + 200 ppm citric extract (Ct); 200 ppm acerola + 200 ppm hydroxytyrosol extract (HXT); 200 ppm acerola + 200 ppm pomegranate extract (P); 200 ppm acerola + 200 ppm rosemary extract (R); Mean ± standard error (M ± s.e.). Different letters (a, b, c) in the same column indicate significant differences between samples (*p* < 0.05). Different letters in the same row (x, y, z) indicate significant differences between samples (*p* < 0.05).

**Table 4:** Physical-chemical quality expressed by changes produced on lipid oxidation (thiobarbituric acid reactive substances (TBARs)) (m ± s.e. mg MDA/kg), protein oxidation (thiol groups concentration) (m ± s.e. mM thiol group/mg protein) and fish degradation (trimethylamine (TMA) and total volatile basic nitrogen (TVB-N)) (m ± s.e. mg/100 g) of fish patties for 14 days under refrigerated storage.

Sample	Days Refrigerated Storage				
	0	4	7	11	14
**TBARs (mg MDA/kg)**					
Control Ct HXT P R	0.3 ± 0.01 ^x^ 0.2 ± 0.01 ^x^ 0.2 ± 0.00 ^x^ 0.2 ± 0.01 ^x^ 0.3 ± 0.01 ^x^	0.6 ± 0.02 ^ay^ 0.6 ± 0.01 ^az^ 0.4 ± 0.01 ^bcy^ 0.3 ± 0.01 ^cy^ 0.5 ± 0.01 ^byz^	0.6 ± 0.01 ^ay^ 0.6 ± 0.01 ^ayz^ 0.5 ± 0.00 ^bcyz^ 0.4 ± 0.01 ^cy^ 0.5 ± 0.01 ^bcyz^	0.6 ± 0.01 ^by^ 0.7 ± 0.01 ^ayz^ 0.6 ± 0.01 ^bcz^ 0.5 ± 0.01 ^cyz^ 0.5 ± 0.01 ^cyz^	0.8 ± 0.01 ^az^ 0.7 ± 0.01 ^byz^ 0.6 ± 0.01 ^cz^ 0.6 ± 0.01 ^cz^ 0.6 ± 0.00 ^cz^
**Thiol groups concentration (mmol thiol/mg protein)**					
Control Ct HXT P R	37.5 ± 0.1 ^w^ 32.9 ± 0.0 ^w^ 34.7 ± 0.1 ^w^ 32.0 ± 0.0 ^w^ 32.6 ± 0.0 ^w^	32.0 ± 0.0 ^x^ 21.4 ± 0.0 ^x^ 18.6 ± 0.1 ^x^ 26.8 ± 0.0 ^x^ 27.0 ± 0.0 ^x^	26.7 ± 0.0 ^xy^ 16.4 ± 0.1 ^xy^ 17.2 ± 0.1 ^xy^ 24.1 ± 0.1 ^xy^ 18.1 ± 0.0 ^xy^	15.7 ± 0.0 ^yz^ 15.4 ± 0.0 ^yz^ 18.6 ± 0.0 ^yz^ 20.7 ± 0.1 ^yz^ 13.5 ± 0.4 ^yz^	10.1 ± 0.0 ^z^ 12.9 ± 0.1 ^z^ 14.7 ± 0.1 ^z^ 16.7 ± 0.0 ^z^ 11.0 ± 0.0 ^z^
**TMA**					
Control Ct HXT P R	1.0 ± 0.0 ^z^ 0.5 ± 0.0 ^z^ 0.2 ± 0.1 ^z^ 0.9 ± 0.0 ^z^ 0.6 ± 0.1 ^z^	4.5 ± 0.0 ^yz^ 4.7 ± 0.0 ^yz^ 4.4 ± 0.0 ^yz^ 1.5 ± 0.2 ^yz^ 3.5 ± 0.0 ^yz^	7.8 ± 0.0 ^y^ 7.0 ± 0.1 ^y^ 6.3 ± 0.0 ^y^ 2.9 ± 0.0 ^y^ 5.1 ± 0.1 ^y^	17.3 ± 0.0 ^x^ 16.1 ± 0.0 ^x^ 10.8 ± 0.0 ^x^ 7.1 ± 0.0 ^x^ 9.2 ± 0.1 ^x^	18.3 ± 0.0 ^x^ 17.3 ± 0.0 ^x^ 15.4 ± 0.1 ^x^ 10.7 ± 0.0 ^x^ 11.7 ± 0.0 ^x^
**TVB-N**					
Control Ct HXT P R	4.0 ± 0.1 ^z^ 5.2 ± 0.1 ^z^ 4.6 ± 0.0 ^z^ 4.4 ± 0.1 ^z^ 4.8 ± 0.0 ^z^	32.2 ± 0.1 ^z^ 23.9 ± 0.1 ^z^ 27.9 ± 0.0 ^z^ 7.5 ± 0.0 ^z^ 14.2 ± 0.0 ^z^	87.9 ± 0.2 ^y^ 81.8 ± 0.1 ^y^ 50.6 ± 0.1 ^y^ 12.4 ± 0.1 ^y^ 42.2 ± 0.1 ^y^	110.0 ± 0.1 ^x^ 114.4 ± 0.0 ^x^ 108.1 ± 0.0 ^x^ 34.8 ± 0.1 ^x^ 91.1 ± 0.0 ^x^	115.2 ± 0.1 ^x^ 118.4 ± 0.0 ^x^ 120.6 ± 0.0 ^x^ 49.7 ± 0.0 ^x^ 100.9 ± 0.0 ^x^

Control: 200 ppm acerola + 200 ppm citric extract (Ct); 200 ppm acerola + 200 ppm hydroxytyrosol extract (HXT); 200 ppm acerola + 200 ppm pomegranate extract (P); 200 ppm acerola + 200 ppm rosemary extract (R); Mean ± standard error (M ± s.e.). Different letters (a, b, c) in the same column indicate significant differences between samples (*p* < 0.05). Different letters (w, x, y, z) in the same row indicate significant differences between samples (*p* < 0.05).

**Table 5:** Volatile compounds (AU × 10^−6^ per g dry mater) (mean area ± standard deviation) of fish patty samples for 11 days under refrigerated storage.

Volatile Compounds	Sample	Day 0	Day 11	Volatile Compounds	Sample	Day 0	Day 11
Spices				Microbiological degradation			
Eucalyptol	Control	0.66 ± 0.01 ^b^	0.56 ± 0.02 ^b^	Propanoic acid	Control	nd	2.05 ± 0.01
	Ct	9.25 ± 0.05 ^a^	3.24 ± 0.06 ^a^		Ct	nd	3.43 ± 0.02
	R	10.74 ± 0.10 ^a^	5.80 ± 0.02 ^a^		R	nd	3.26 ± 0.02
	P	nd	nd		P	nd	4.01 ± 0.02
	HXT	nd	nd		HXT	nd	2.35 ± 0.01
Limonene	Control	0.80 ± 0.02 ^cd^	0.90 ± 0.01 ^cd^	Malonic acid	Control	3.54 ± 0.02 ^b^	3.01 ± 0.03 ^b^
	Ct	2.80 ± 0.01 ^a^	2.25 ± 0.01 ^a^		Ct	3.61 ± 0.03 ^b^	3.53 ± 0.04 ^b^
	R	1.52 ± 0.01 ^b^	1.27 ± 0.01 ^b^		R	2.05 ± 0.03 ^b^	2.48 ± 0.03 ^b^
	P	1.10 ± 0.03 ^bc^	1.39 ± 0.01 ^bc^		P	6.91 ± 0.08 ^a^	6.84 ± 0.08 ^a^
	HXT	0.78 ± 0.01 ^d^	0.33 ± 0.01 ^d^		HXT	7.44 ± 0.09 ^a^	4.79 ± 0.05 ^a^
Fish degradation							
Methylamine	Control	1.14 ± 0.02	9.55 ± 0.02	Toluene	Control	1.52 ± 0.01	0.91 ± 0.01
	Ct	1.85 ± 0.02	10.93 ± 0.2		Ct	4.94 ± 0.05	2.00 ± 0.02
	R	1.75 ± 0.02	5.60 ± 0.05		R	3.15 ± 0.01	1.77 ± 0.02
	P	1.29 ± 0.02	1.96 ± 0.01		P	4.77 ± 0.02	2.52 ± 0.01
	HXT	2.11 ± 0.03	7.67 ± 0.06		HXT	5.56 ± 0.04	1.97 ± 0.02
Lipid autooxidation				Sea Pollution Agents			
1-butanol	Control	nd	6.93 ± 0.03 ^e^	Arsenous acid	Control	6.59 ± 0.04	7.57 ± 0.01
	Ct	nd	15.25 ± 0.10 ^c^		Ct	8.88 ± 0.06	4.76 ± 0.05
	R	nd	17.16 ± 0.20 ^b^		R	6.55 ± 0.05	3.46 ± 0.02
	P	nd	23.26 ± 0.20 ^a^		P	8.31 ± 0.05	4.77 ± 0.03
	HXT	nd	13.06 ± 0.12 ^d^		HXT	7.87 ± 0.08	4.78 ± 0.04
2-nonanone	Control	nd	nd	Furan	Control	0.29 ± 0.01 ^b^	0.16 ± 0.01 ^b^
	Ct	2.23 ± 0.01 ^ab^	1.13 ± 0.01 ^ab^		Ct	0.37 ± 0.01 ^b^	0.22 ± 0.01 ^b^
	R	1.50 ± 0.02 ^b^	0.98 ± 0.01 ^b^		R	0.33 ± 0.01 ^b^	0.16 ± 0.01 ^b^
	P	2.18 ± 0.03 ^a^	1.81 ± 0.01 ^a^		P	0.51 ± 0.01 ^a^	0.46 ± 0.01 ^a^
	HXT	1.24 ± 0.02 ^ab^	1.33 ± 0.03 ^ab^		HXT	0.47 ± 0.01 ^ab^	0.27 ± 0.01 ^ab^

Control: 200 ppm acerola + 200 ppm citric extract (Ct); 200 ppm acerola + 200 ppm hydroxytyrosol extract (HXT); 200 ppm acerola + 200 ppm pomegranate extract (P); 200 ppm acerola + 200 ppm rosemary extract (R); Mean ± standard error (M ± s.e.). nd: undetected. Different letters (a, b, c, d, e) in the same column indicate significant differences between samples (*p* < 0.05).

**Table 6:** Ingredients (g) of reformulated fish patty samples.

Ingredients	Control	Ct	HXT	P	R
Hake (g)	852	852	852	852	852
Water (ml)	100	100	100	100	100
Commercial mix (g)	48				
Salt (g)		19	19	19	19
Fibers (g)		25	25	25	25
Soy albumin (g)		14	14	14	14
Essential oils (ml):					
Ecoflax ^®^		5.7	5.7	5.7	5.7
Biomega Tech Algae ^®^		5.7	5.7	5.7	5.7
Natural extracts (ppm):					
Acerola		200	200	200	200
Ct		200			
HXT			200		
P				200	
R					200

Commercial mix composed of vegetable fibers, salt, potato starch, stabilizer (processed Eucheuma seaweed (PES) E-407-a), acidity correctors (sodium citrate E-331 and sodium acetate E-262), spices, spice extracts, and antioxidant (sodium ascorbate E-301). Citric extract (Ct); hydroxytyrosol extract (HXT) obtained from vegetable waters of olive tree; pomegranate extract (P), rosemary extract (R). Essential oils Ecoflax^®^ and Biomega Tech Algae^®^ were rich in 40% α-linolenic acid and 40% docosahexaenoic acid, respectively.
